# Annexin-A1: Therapeutic Potential in Microvascular Disease

**DOI:** 10.3389/fimmu.2019.00938

**Published:** 2019-04-30

**Authors:** Gareth S. D. Purvis, Egle Solito, Christoph Thiemermann

**Affiliations:** ^1^William Harvey Research Institute, Queen Mary University of London, London, United Kingdom; ^2^Sir William Dunn School of Pathology, University of Oxford, Oxford, United Kingdom

**Keywords:** Annexin-A1 (ANXA1), diabetes, inflammation, tissue protection, signaling

## Abstract

Annexin-A1 (ANXA1) was first discovered in the early 1980's as a protein, which mediates (some of the) anti-inflammatory effects of glucocorticoids. Subsequently, the role of ANXA1 in inflammation has been extensively studied. The biology of ANXA1 is complex and it has many different roles in both health and disease. Its effects as a potent endogenous anti-inflammatory mediator are well-described in both acute and chronic inflammation and its role in activating the pro-resolution phase receptor, FPR2, has been described and is now being exploited for therapeutic benefit. In the present mini review, we will endeavor to give an overview of ANXA1 biology in relation to inflammation and functions that mediate pro-resolution that are independent of glucocorticoid induction. We will focus on the role of ANXA1 in diseases with a large inflammatory component focusing on diabetes and microvascular disease. Finally, we will explore the possibility of exploiting ANXA1 as a novel therapeutic target in diabetes and the treatment of microvascular disease.

Annexin-A1 (ANXA1) is a 37 kDa phospholipid-binding protein widely expressed in many tissues including leukocytes, lymphocytes, epithelial cells, and endothelial cells. ANXA1 is present intracellularly and at the membrane (1), but can also be secreted into the circulation were it can signal in both an autocrine and paracrine manner (2, 3). However, in disease ANXA1 levels are modulated most notably when endogenous glucocorticoids levels are altered. Patients with Addison's disease exhibit lower levels of ANXA1 in leukocytes due to reduced cortisol production. In contrast, patients with Cushing's syndrome have elevated levels of ANXA1 secondary to excessive cortisol production (4).

## Regulation an Expression and Secretion of ANXA1

The role ANXA1 plays as an anti-inflammatory as a molecule up-regulated by glucocorticoids was first described over three decades ago (5–7). ANXA1 expression was shown to be higher in alveolar macrophages, obtained by broncho-alveolar lavage, from patients receiving glucocorticoid treatment for inflammatory lung disease (8). Glucocorticoid administration to peripheral blood mononuclear (PBMN) cells increased ANXA1 expression in both a temporal and dose-dependent manner (9, 10). ANXA1 is upregulated by both IL-6 and phorbol-myristate (PMA) in human lung epithelial cell line A549 via activation of transcription factor C/EBPβ (11). IL-6, dexamethasone and endothelial cell adhesion cause ANXA1 to be trafficked to the cell surface and to be secreted, which has important implications for ANXA1 mediated anti-inflammatory actions of glucocorticoids (7, 12, 13).

**Figure 1:**
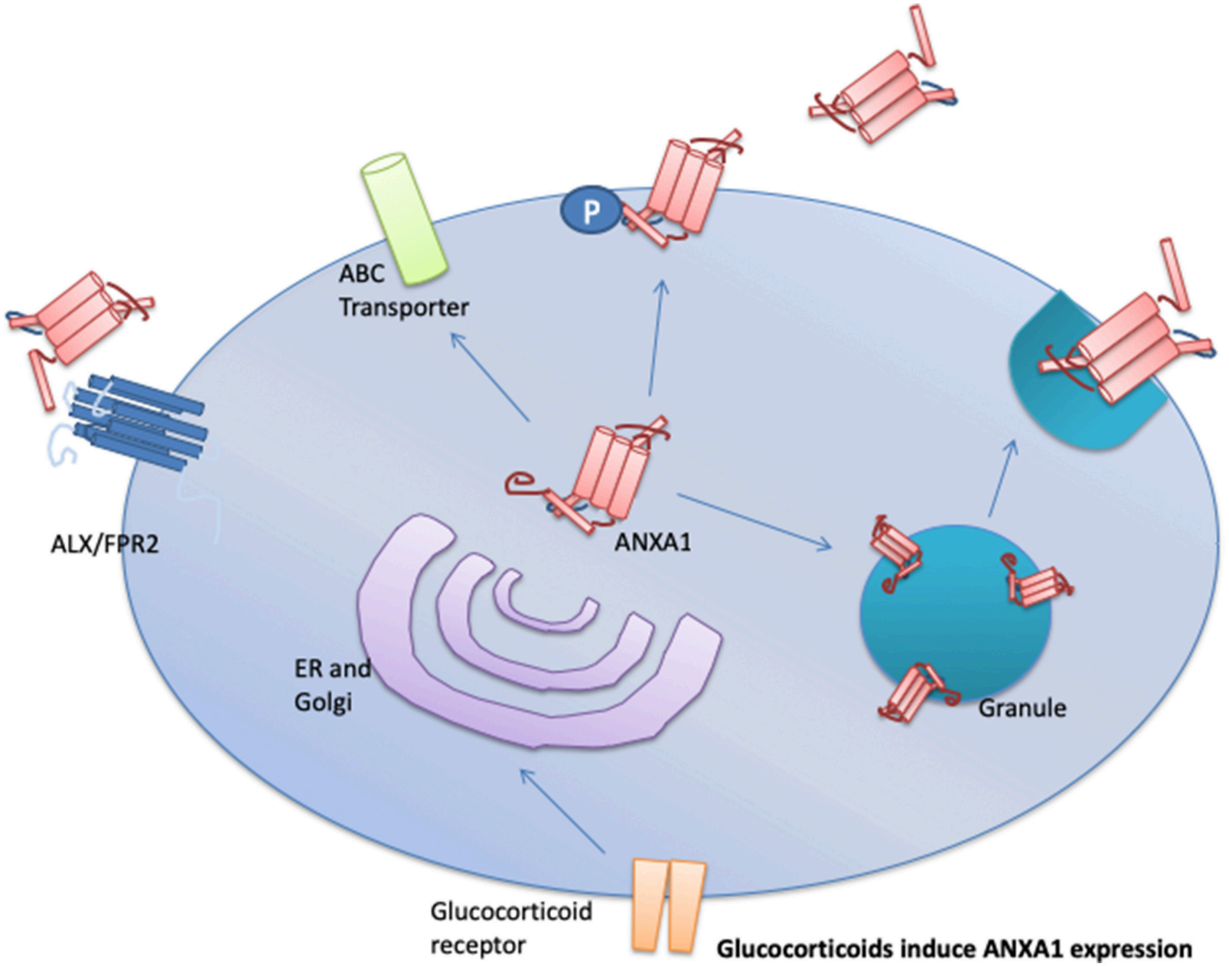
Upon cellular activation, ANXA1 is mobilized to the plasma membrane and then secreted in one of three mechanisms depending in the cell type involved. These mechanisms are: (1) through the ATP-binding (ABC) transporter; (2) via direct phosphorylation of ANXA1 on serine-27 followed by membrane localization to the plasma membrane, and (3) fusion of ANXA1 loaded granules to the plasma membrane. Once released ANXA1 can act in an autocrine, paracrine, and juxtacrine manner to activate ALX/FPR2 signaling.

The secretion of ANXA1 occurs in one of three ways; (i) via the ATP-binding cassette transport system (14), (ii) phosphorylation of ANXA1 on serine-27 in pituitary cells (15), and (iii) it is released from gelatinase granules following cellular exposure to weak activating signals i.e., cell adhesion to endothelium (Figure 1) (16, 17). Once transported to the outside of the plasma membrane, ANXA1 can be tethered in a calcium-dependent manner. Under resting conditions, the N-terminus of ANXA1 is buried in a pocket. Extracellular Ca^2+^ concentrations of ≥1 mM are needed to facilitate the release of the N-terminal region allowing ANXA1 to aggregate at the membrane (18, 19); where it can signal in both an autocrine or paracrine fashion (17). ANXA1 can be cleaved into a 33 kDa fragment by elastases, metalloproteases, or proteinase 3 (20–22). This N-terminal fragment is biologically active; however, it is some 20-fold less potent than the un-cleaved full length protein. Ac2-26 which is a synthetic mimetic of the N-terminal fragment requires 14 time more in terms of molarity to elicit the same levels of gene expression changes as the full length protein (12, 23). Therefore, it is still unclear if (a) the cleavage produces the active form from the full protein, whereby ANXA1 is a pro-protein, (b) a bioactive fragment is produced, or (c) cleavage promotes homeostasis and limits the actions of ANXA1. Pederzoli-Ribeil et al. report that cleavage resistant forms of ANXA1 and its peptide Ac2-26 demonstrate prolonged biological function (22).

## ANXA1 and Formyl Peptide Receptor 2 (ALX/FPR2)

The effects of ANXA1 are mediated by the N-terminal interaction with the G-protein coupled formyl peptide receptor-2 (ALX/FPR2) (24). Like ANXA1, the ALX/FRP2 receptor is expressed on a wide variety of cell types including fibroblasts, endothelial cells, and stromal cells; but its expression is most abundant on leukocytes (25). The ALX/FPR2 receptor is coupled to both Gα and Gβγ. The signaling cascades that are activated by different ALX/FPR2 agonists have been reviewed in detail by Cattaneo et al. (26).

The generation of the ANXA1 knock out mouse has allowed the design of many studies, which have resulted in a better understanding of ANXA1 biology (27). ANXA1 knock out mice are resistant to the effects of glucocorticoids in a model of amatory arthritis of suggesting that ANXA1 is one of the main anti-inflammatory effector molecules of glucocorticoids (28). However, gaining a better understanding of the role of FPR2 on the anti-inflammatory effects of ANXA1 has been more challenging. Humans have three FPR genes, while mice have at least eight FPR genes, with a high degree of sequence homology between members in mice. Thus, although there is very good evidence that activation of FPR2 mediates the anti-inflammatory effects of ANXA1 in humans, this is less clear in mice, as in this species ANXA1 can activate both FPR1 and FPR2 (29). Indeed, Cooray et al. demonstrated for the first time that agonist binding and dimerization state contributed to the conformational landscape of FPR's and allowed them to elicit various pro-resolution functions (30).

As aforementioned many of the biological actions of ANXA1 can be mimicked using the peptide Ac2-26 (31). Hayhoe et al. demonstrate that blocking FPR2/ALXR with a monoclonal antibody prevented ANXA1/Ac2-26 induced inhibition of human neutrophil transmigration and adhesion to the endothelial-cell monolayers under flow conditions (32).

## ANXA1: an Anti-inflammatory Mediator

ANXA1 is best known for its anti-inflammatory and pro-resolving properties. Known mechanisms of action span from the inhibition of pro-inflammatory mediators release (PGE_2_ and leukotrines) (33), to tissue repair (34), and to the blockade of leukocyte migration through an inflamed endothelium (35, 36). ANXA1 induces L-selectin shedding on neutrophils and the detachment of monocytic cells from the endothelium by reducing α4β1 integrin clustering and activation (12). Treatment of human neutrophils with Ac2-26 (a synthetic N-terminal fragment of ANXA1) reduces their ability to adhere and undergo chemotaxis (37, 38). In a zymosan-induced peritonitis model, ANXA1^−/−^ mice display a lower degree of PMN recruitment compared to wild-type littermates (39). Analysis of the cremaster muscle microcirculation by intravital microscopy after stimulation with zymosan showed increased leukocyte emigration, but not rolling or adhesion in ANXA1^−/−^ (29, 35). Similarly, ALX/FPR2^−/−^ mice exhibit reduced leukocytes trafficking and emigration after mesenteric ischemia reperfusion (29). ANXA1 also suppresses indomethacin-induced leukocyte adherence to the vascular endothelium (40).

ANXA1 also aids resolution of inflammation through accelerating apoptosis. Transfection of monocytic U937 cells with full length recombinant ANXA1 constitutively activates caspase-3 activity (41). Moreover, ANXA1 stimulates increased cytosolic calcium flux resulting in the de-phosphorylation of the Bcl-2 associated death promoter (Bad), thus, activating the apoptotic effector machinery (15). McArthur et al. discovered a novel mechanism whereby ANXA1 is released by apoptotic neutrophils targeting only them for phagocytosis by recruited monocytes, thus protecting the surrounding healthy tissue from damage (42). ANXA1^−/−^ mice provided further evidence for a functional role of ANXA1 in efferocytosis, whereby, bone marrow derived macrophages (BMDM) from ANXA1^−/−^ mice were inefficient in the clearance of apoptotic cells (43). Dalli et al. confirmed that ANXA1 expression in BMDM was needed for the recognition and efferocytosis of apoptotic neutrophils (44).

### Central Nervous System

ANXA1 plays a pivotal role in maintaining blood brain barrier integrity. ANXA1 is highly expressed at the site of cell-cell contacts specifically, at tight junctions. ANXA1^−/−^ showed a down modulation and alteration in tight junction protein occludin and VE-cadherin (2, 45). Cristante et al. demonstrated that ANXA1 signaling through FRP2/ALX inhibits the small GTPase RhoA, allowing for actin stabilization. Under physiological conditions, *ANXA1*^−/−^mice exhibit an increase in permeability of the blood brain barrier as measured by MRI, increases leaking of Evans blue extravasation and serum IgG (2). Taken together, these findings suggest that ANXA1 plays a key role in the tightness of the blood brain barrier.

Patients with multiple sclerosis have decreased expression of ANXA1 in brain parenchymal capillaries (2). Interestingly, the loss of ANXA1 expression occurred at sites distant from the active lesion. Enhanced ANXA1 expression has also been documented in macrophages and perivascular lymphocytes at sights of active lesions (46). One could speculate that an increase in the permeability of the blood brain barrier triggers the infiltration of immune cells into an immune privileged area, which in turn exacerbates the disease. Ries et al. demonstrated ANXA1 expression is increased both in the brain of patients with Alzheimer's disease and animal models of Alzheimer's disease at early stages of the disease. They report that ANXA1 regulates amyloid-β phagocytosis in microglia by increasing its enzymatic degradation by neprilysin (47). Such apparent contradictory data shows that ANXA1 plays differing roles in these two diseases. From one side the down regulation of ANXA1 is important component of the BBB allowing for increased leakage allowing T cells that have lost a self-recognition to pass in the parenchyma and attack the myelin sheet. On the other hand ANXA1 upregulation at microglia level in human and animal model of Alzheimer's disease demonstrates that ANXA1 has anti-inflammatory effects in control the M1 to M2 phenotypic switch of microglia M1/M2 (48).

### Cardiovascular Disease

Kusters et al. demonstrated that hrANXA1 treatment of LDLR^−/−^ mice reduces atherosclerotic plaque burden (49). In contrast, atherogenic ALX/FPR2^−/−^ mice develop less lipid streaks in the descending aorta (50). Drechsler et al. also demonstrate that Ac2-26 (a synthetic peptide of the last 25 amino acids of the N-terminal fragments of AXNA1) inhibits CCL5-induced conformation change of β2 integrin into its activate state in neutrophils and monocytes, resulting in a reduction in early atherogenesis and plaque formation (51). These studies further highlight the therapeutic potential of ANXA1 in atherosclerosis an important co-morbidity of diabetes. Fredman et al. used collagen IV-targeting nanoparticles containing Ac2-26 as a therapeutic intervention in atherogenic LDLR^−/−^ mice. Interestingly, Ac2-26 was released from the nanoparticles and accumulated in vessel lesions, increasing plaque stability (52).

ANXA1 has been shown to be tissue protective in arterial wall healing after wire injury in ApoE^−/−^ mice; limiting arterial neointima formation by reducing macrophage proliferation (53). Gobbetti et al. demonstrated a non-redundant role for the ALX/FPR2 axis in polymicrobial sepsis. The observed protective effects of Ac2-50 (a synthetic peptide of the last 28 amino acid of the N-terminal fragment of ANXA1) were lost in FRP2/3^−/−^ mice suggesting that activation by Ac2-50 of the ALX/FPR2 confers tissue protection (54). In patients with stable coronary artery disease, Bergström et al. describe that ANXA1 located on the surface of peripheral monocytes serves as a marker of glucocorticoid sensitivity, thereby reflecting the anti-inflammatory capacity of these cells (55).

### Ischemia Reperfusion Injury

ANXA1 and its N-terminal peptides are protective in various models of ischemia reperfusion injury (IRI) (56). D'Amico et al. demonstrated a decrease in myocardial infarct size upon infusion of recombinant ANXA1 (57). Treatment with Ac2-26 decreased infarct size and reduced MPO and IL-1β content in infarcted hearts (58). The peptide Ac2-26 preserved cardiomyocyte contractility by activating PKC, p38, and K_ATP_ channels (59). Compound 17b, a biased agonist of FPR1/2, attenuates both early and late inflammatory responses associated with reperfusion after an acute myocardial infarction (60). Intracerebroventricular administration of Ac2-26 reduces stroke volume (infarct size) and cerebral edema in rats (61), possibly by reducing leukocyte-endothelial interactions (62). Vital et al. demonstrated that Ac2-26 attenuated neutrophil and platelet activation and neutrophil–platelet aggregation in the murine cerebral microvasculature after induction of cerebral ischemia-reperfusion injury in mice (63). Interestingly, ANXA1 translocates to the nucleus to activate pro-inflammatory gene expression in microglial cells in an *in vitro* model of ischemia-reperfusion injury (64). McArthur et al. demonstrated that FPR2/ALX^−/−^mice had greater BBB leakage post-ischemia than wild type littermates (45).

## ANXA1 in Diabetes

Early work by Melki et al. demonstrated that physiological concentrations of ANXA1 had the ability to inhibit tyrosine 21 phosphorylation on the insulin receptor; which is needed for insulin secretion (65). A later study found that ANXA1 increased insulin secretion in rat pancreatic and MIN6N8s cells by cell surface binding; although the receptor is not described (66). Furthermore, ANXA1 was demonstrated to be serine phosphorylated upon exposure to high glucose levels (66), suggesting, phosphorylation is necessary to induce glucose stimulated insulin release. However, ANXA1^−/−^ mice display no augmentation in oral glucose tolerance test (*in vivo*), and *ex vivo* isolated pancreas from ANXA1^−/−^ mice have the same level glucose of stimulated insulin secretion as WT mice, suggesting, that ANXA1 is not essential for insulin secretion to lower blood glucose levels (67). Yet under hyperglycemic conditions, ANXA1^−/−^ mice fed a HFD demonstrate increased inhibitory phosphorylation on IRS-1 indicative of severe insulin resistance (68, 69). And when ANXA1^−/−^ mice were treated with streptozotocin (STZ) to induce experimental type-1 diabetes they displayed a more severe augmentation in oral glucose tolerance test (OGTT) compared to WT mice (3). Similarly, when ANXA1^−/−^ mice are fed a HFD they develop a more severe diabetic phenotype, characterized by increased blood glucose levels, elevated insulin levels and more severe augmentation in OGTT (69–71). Taken collectively, these lines of evidence suggest, but not prove that ANXA1 is important in the regulation of glucose levels in diseased state but may have a redundancy in health. Cristante et al. demonstrated that ANXA1 directly interacts with and regulates RhoA in endothelial cells (2), while ANXA1^−/−^ mice have constitutively activated RhoA in the kidney and liver (69). Subsequent work by Purvis et al. revealed a mechanistic link between ANXA1 expression, RhoA and IRS-1 in diabetic mice (68, 69, 72).

Numerous reports have demonstrated that protein expression of ANXA1 is decreased in diabetes (69, 71). It is currently unclear the mechanism by which this occurs, one possibility is that the rate of secretion is increased, or there could be transcriptional regulation at the transcript level. We and others have shown that the plasma levels of ANXA1 are elevated in patients with long-standing type-1 diabetes (over 25 years from diagnosis) and those with type-2 diabetes and obesity compared to age-matched healthy controls (3, 69, 73). Murine models of diabetes have demonstrated a similar rises in ANXA1 levels in serum as seen in humans with diabetes. However, the biological consequence of elevated ANXA1 in diabetes remains unclear. One possible reason is that ANXA1 is secreted from tissues under hyperglycemic and hyperlipidemic conditions, however, further research is needed to confirm this. Interesting, serum ANXA1 levels in patients with type 1 and type 2 diabetes did not correlate with increased systemic inflammation (C-reactive protein levels) (3, 69). ANXA1 also been has been shown to be a good diagnostic marker of glomerular injury and in particular diabetic nephropathy (74).

### ANXA1 in Diabetic Nephropathy

In addition to aberrant glucose handling, patients with diabetes will develop overt or sub-clinical microvascular complications (diabetic nephropathy, neuropathy, and retinopathy) over the course of their disease. These complications occur predominantly in tissues where glucose uptake is insulin independent (kidney, retina, and the endothelium) as these tissues are exposed to glucose levels close to blood glucose levels. ANXA1^−/−^ mice have more severe diabetic nephropathy compared to WT littermates in STZ-induced experimental type 1 diabetes and in a model of HFD induced insulin resistance. In both models, mice developed more severe proteinuria and had more pronounced loss of brush borders in the S1-S2 segment of the proximal convoluted tubules (3, 69).

The natural history of diabetic nephropathy comes as a result of the combined effects of direct glucose mediated endothelial damage, superoxide production, and advance glycation end-products due to prolonged hyperglycemia (75). Hyperglycemia triggers the production of excessive reactive oxygen species (ROS) leading to oxidative stress, primarily in the blood vessels, which can ultimately lead to endothelial senescence an early sign of vascular complications in diabetes (76, 77). Over production of ROS leads to the uncoupling of endothelial nitric oxide synthase (eNOS) leading to reduced nitric oxide production, which impairs endothelial dependent vasodilatation, ultimately leading to an increase in blood pressure. ANXA1^−/−^ mice fed a HFD show a decreased eNOS activity in the kidney, which can be restored by treatment with human recombinant ANXA1 (69). Elevated blood pressure is a common co-morbidity seen in patients with diabetes. Regulation of blood pressure is leading cause of kidney dysfunction; most therapies for the management of diabetic nephropathy revolve around normalizing blood pressure using angiotensin-converting enzymes inhibitors and angiotensin receptor blockers. Jelminic et al. demonstrate that ANXA1^−/−^ mice with type-2 diabetes display more severe cardiac remodeling, coupled with increased vascular compliance an early sign of hypertension compared with their wild-type littermates (78). ANXA1^−/−^ mice also have constitutively activated MYPT1 (69), which regulates the contraction and relaxation of vascular smooth muscle and maintains blood pressure (79).

The development of renal fibrosis is a classical hallmark of the diabetic kidney. ANXA1^−/−^ with STZ induced type-1 diabetes develop more renal fibrosis compared to diabetic WT mice. Additionally, treatment with hrANXA1 could reduce renal fibrosis in type-1 diabetic mice, by inhibiting ERK1/2, p38, and JNK (3). Neymeyer et al. also demonstrated in a model of hypertensive nephropathy that ANXA1 signaling had anti-fibrotic effects on renal fibroblasts via ALX/FPR2 (80). Some of the anti-fibrotic and reno-protective effects of the ANXA1/FPR2 axis have been attributed to the accumulation of anti-inflammatory M2 macrophages in the nephron; an effect which was first demonstrated in an a model of acute anti-Thy1.1 nephritis (81). Further work is needed to determine if similar phenotypic class switching of macrophages occurs in the diabetic kidney.

### ANXA1 as a Potential Therapeutic Tool in Diabetes

Human recombinant ANXA1 and the N-terminal peptide (Ac2-26) have both been used as pharmacological tools in many *in vivo* models of both acute and chronic inflammation (3, 49, 54, 69, 82, 83). However, the concentration of Ac2-26 needed to elicit the same biological effect as full length ANXA1 are some 20 times higher (12). Many of the effects of ANXA1 and its peptides have are mediated by ALX/FPR2 axis, however, there is much evidence provided for FPR1 facilitating wound healing and tissue protective effects of ANXA1 in the epithelium (34).

STZ-induced type-1 diabetic mice treated with human recombinant ANXA1 do not develop microvascular complications (diabetic cardiomyopathy and nephropathy) even though they have elevated blood glucose (3), suggesting a key organ protection effect independent of glucose lowering. In the same model mice given hrANXA1 therapeutically (after microvascular complications had developed) did not display a further decline in cardiac and renal function seen in vehicle treated mice. The therapeutic benefits in a pre-clinical model of diabetes were in part mediated by restoration of the pro-survival and tissue protective Akt and MAPK pathways (3, 84). In line with these findings, ANXA1 has been shown to restore ERK1/2 and Akt signaling in murine models of cardiac ischemia reperfusion (60), suggesting that ANXA1 is a key regulator of both pro-survival and anti-inflammatory pathways. Independently, Yoon et al. demonstrated that mice fed a HFD and treated with the N-terminal fragment of ANXA1 (Ac2-26) developed less severe insulin resistance resulting in reduced hyperglycemia, and reactivating Akt activity (71). Activation of the small GTPase RhoA is a validated target for the treatment of microvascular complications, treatment of hrANXA1 to mice fed a HFD reduced RhoA activity and protected the kidney and liver from functional decline (69).

## Perspectives and Concluding Remarks

The discovery of ANXA1, over 20 years ago, as an endogenous anti-inflammatory mediator of glucocorticoids generated a lot of interest both in academia and in the pharmaceutical industry. Although many preclinical data demonstrate good efficacy of ANXA1 or its peptides in many diseases associated with acute or chronic inflammation, translational (human) studies have yet to be performed. In diabetes, the evidence suggests that endogenous ANXA1 may be a key player in regulating insulin secretion. When given therapeutically, ANXA1 protects peripheral organs against the injury and dysfunction caused by hyperglycemia or, indeed, hyperlipidemia. More work, however, is needed to elucidate the mechanisms behind the beneficial effects of ANXA1 in preventing the microvascular complications associated with diabetes.

## Author Contributions

All authors listed have made a substantial, direct and intellectual contribution to the work, and approved it for publication.

### Conflict of Interest Statement

The authors declare that the research was conducted in the absence of any commercial or financial relationships that could be construed as a potential conflict of interest.

## References

[1] Goulding N Godolphin JL Sharland PR Maddison PJ Sampson M Peers SH. Anti-inflammatory lipocortin 1 production by peripheral blood leucocytes in response to hydrocortisone. Lancet. (1990) 335:1416–8. 10.1016/0140-6736(90)91445-G 1972208

[2] Cristante E McArthur S Mauro C Maggioli E Romero IA Wylezinska-Arridge M. Identification of an essential endogenous regulator of blood-brain barrier integrity, and its pathological and therapeutic implications. Proc Natl Acad Sci USA. (2013) 110:832–41. 10.1073/pnas.1209362110 23277546 PMC3549094

[3] Purvis GSD Chiazza F Chen J Azevedo-Loiola R Martin L Kusters DHM. Annexin A1 attenuates microvascular complications through restoration of Akt signalling in a murine model of type 1 diabetes. Diabetologia. (2017) 61:482–95 10.1007/s00125-017-4469-y 29085990 PMC6448955

[4] Mulla A Leroux C Solito E Buckingham JC. Correlation between the antiinflammatory protein annexin 1 (lipocortin 1) and serum cortisol in subjects with normal and dysregulated adrenal function. J Clin Endocrinol Metab. (2005) 90:557–62. 10.1210/jc.2004-1230 15509636

[5] Cirino G Flower RJ. Human recombinant lipocortin 1 inhibits prostacyclin production by human umbilical artery *in vitro*. Prostaglandins. (1987) 34:59–62. 10.1016/0090-6980(87)90262-0 2961007

[6] Flower RJ Rothwell NJ. Lipocortin-1: cellular mechanisms and clinical relevance. Trends Pharmacol Sci. (1994) 15:71–6. 8184489 10.1016/0165-6147(94)90281-x

[7] Solito E de Coupade C Parente L Flower RJ Russo-Marie F. IL-6 stimulates Annexin 1 expression and translocation and suggests a new biological role as a class II acute phase protein. Cytokine. (1998) 10:514–21. 10.1006/cyto.1997.0325 9702415

[8] De Caterina R Sicari R Giannessi D Paggiaro PL Paoletti P Lazzerini G. Macrophage-specific eicosanoid synthesis inhibition and lipocortin-1 induction by glucocorticoids. J Appl Physiol. (1993) 75:2368–75. 8125852 10.1152/jappl.1993.75.6.2368

[9] Sawmynaden P Perretti M. Glucocorticoid upregulation of the annexin-A1 receptor in leukocytes. Biochem Biophys Res Commun. (2006) 349:1351–5. 10.1016/j.bbrc.2006.08.179 16973129

[10] Solito E Mulla A Morris JF Christian HC Flower RJ Buckingham JC. Dexamethasone induces rapid serine-phosphorylation and membrane translocation of annexin 1 in a human folliculostellate cell line via a novel nongenomic mechanism involving the glucocorticoid receptor, protein kinase C, phosphatidylinositol 3-kinase, and mitogen-activated protein kinase. Endocrinology. (2003) 144:1164–74. 10.1210/en.2002-220592 12639897

[11] Solito E de Coupade C Parente L Flower RJ Russo-Marie F. Human annexin 1 is highly expressed during the differentiation of the epithelial cell line A 549: involvement of nuclear factor interleukin 6 in phorbol ester induction of annexin 1. Cell Growth Differ. (1998) 9:327–36. 9563852

[12] Solito E Romero IA Marullo S Russo-Marie F Weksler BB. Annexin 1 binds to U937 monocytic cells and inhibits their adhesion to microvascular endothelium: involvement of the 4 1 integrin. J Immunol. (2000) 165:1573–81. 10.4049/jimmunol.165.3.1573 10903766

[13] Perretti M Croxtall JD Wheller SK Goulding NJ Hannon R Flower RJ. Mobilizing lipocortin 1 in adherent human leukocytes downregulates their transmigration. Nat Med. (1996) 2:1259–1262. 10.1038/nm1196-1259 8898757

[14] Wein S Fauroux M Laffitte J de Nadaï P Guaïni C Pons F. Mediation of annexin 1 secretion by a probenecid-sensitive ABC-transporter in rat inflamed mucosa. Biochem Pharmacol. (2004) 67:1195–202. 10.1016/j.bcp.2003.11.015 15006554

[15] Solito E Kamal A Russo-Marie F Buckingham JC Marullo S Perretti M. A novel calcium-dependent proapoptotic effect of annexin 1 on human neutrophils. FASEB J. (2003) 17:1544–6. 10.1096/fj.02-0941fje 12824302

[16] Perretti M Christian H Wheller SK Aiello I Mugridge KG Morris JF Annexin I is stored within geletinase granules of human neutrophils and mobalised on the cell surface upon adhesion but not phagocytosis. Cell Biol Int. (2000) 24:163–74. 10.1006/cbir.1999.0468 10772777

[17] Perretti M D'Acquisto F. Annexin A1 and glucocorticoids as effectors of the resolution of inflammation. Nat Rev Immunol. (2009) 9:62–70. 10.1038/nri2470 19104500

[18] Rosengarth A Luecke H. A calcium-driven conformational switch of the N-terminal and core domains of annexin A1. J Mol Biol. (2003) 326:1317–25. 10.1016/S0022-2836(03)00027 12595246

[19] Rosengarth A Gerke V Luecke H. X-ray structure of full-length annexin 1 and implications for membrane aggregation. J Mol Biol. (2001) 306:489–98. 10.1006/jmbi.2000.4423 11178908

[20] Rescher U Goebeler V Wilbers A Gerke V. Proteolytic cleavage of annexin 1 by human leukocyte elastase. Biochim Biophys Acta Mol Cell Res. (2006) 1763:1320–4. 10.1016/j.bbamcr.2006.08.041 17023068

[21] Vong L D'Acquisto F Pederzoli-Ribeil M Lavagno L Flower RJ Witko-Sarsat V. Annexin 1 cleavage in activated neutrophils. J Biol Chem. (2007) 282:29998–30004. 10.1074/jbc.M702876200 17681950 PMC2772024

[22] Pederzoli-Ribeil M Maione F Cooper D Al-Kashi A Dalli J Perretti M. Design and characterization of a cleavage-resistant Annexin A1 mutant to control inflammation in the microvasculature. Blood. (2010) 116:4288–96. 10.1182/blood-2010-02-270520 20705760

[23] Rodrigues-Lisoni FC Mehemet DK Peitl P John CD Tajara E Buckingham JC. *In vitro* and *in vivo* studies on CCR10 regulation by Annexin A1. FEBS Lett. (2006) 580:1431–8. 10.1016/j.febslet.2006.01.072 16460738

[24] Perretti M Chiang N La M Fierro IM Marullo S Getting SJ. Endogenous lipid- and peptide-derived anti-inflammatory pathways generated with glucocorticoid and aspirin treatment activate the lipoxin A4 receptor. Nat Med. (2002) 8:1296–1302. 10.1038/nm786 12368905 PMC2777269

[25] Migeotte I Communi D Parmentier M. Formyl peptide receptors: a promiscuous subfamily of G protein-coupled receptors controlling immune responses. Cytokine Growth Factor Rev. (2006) 17:501–19. 10.1016/j.cytogfr.2006.09.009 17084101

[26] Cattaneo F Parisi M Ammendola R. Distinct signaling cascades elicited by different formyl peptide receptor 2 (FPR2) agonists. Int J Mol Sci. (2013) 14:7193–230. 10.3390/ijms14047193 23549262 PMC3645683

[27] Hannon R Croxtall JD Getting SJ Roviezzo F Yona S Pauk-clark MJ Aberrant inflammation and resistance to glucocorticoids in annexin 1 ^−/−^ mouse. FASEB J. (2003) 17:253–5. 10.1096/fj.02-0239fje 12475898

[28] Patel HB Kornerup KN Sampaio AL D'Acquisto F Seed MP Girol AP. The impact of endogenous annexin A1 on glucocorticoid control of inflammatory arthritis. Ann Rheum Dis. (2012) 71:1872–80. 10.1136/annrheumdis-2011-201180 22562975 PMC3440300

[29] Flower D'acquisto RJ Buckingham JC Perretti M Dalli J Patel HB Gray M. Experimental inflammation effects on leukocyte responses and formyl-peptide receptor 2: ligand-specific anti-inflammatory role of the murine. J Immunol Ref. (2010) 184:2611–9. 10.4049/jimmunol.0903526 20107188 PMC4256430

[30] Cooray SN Gobbetti T Montero-Melendez T McArthur S Thompson D Clark AJL. Ligand-specific conformational change of the G-protein-coupled receptor ALX/FPR2 determines proresolving functional responses. Proc Natl Acad Sci USA. (2013) 110:18232–7. 10.1073/pnas.1308253110 24108355 PMC3831442

[31] Perretti M Ahluwalia A Harris JG Goulding NJ Flower RJ. Lipocortin-1 fragments inhibit neutrophil accumulation and neutrophil-dependent edema in the mouse. A qualitative comparison with an anti-CD11b monoclonal antibody. J Immunol. (1993) 151:4306–14. 8409403

[32] Hayhoe RPG Kamal AM Solito E Flower RJ Cooper D Perretti M. Annexin 1 and its bioactive peptide inhibit neutrophil-endothelium interactions under flow: indication of distinct receptor involvement. Blood. (2006) 107:2123–30. 10.1182/blood-2005-08-3099 16278303

[33] Sudlow AW Carey F Forder R Rothwell NJ The role of lipocortin-1 in dexamethasone-induced suppression of PGE2 and TNFα release from human peripheral blood mononuclear cells. Br J Pharmacol. (1996) 117:1449–1456. 10.1111/j.1476-5381.1996.tb15305.x 8730738 PMC1909467

[34] Leoni G Nusrat A. Annexin A1: shifting the balance towards resolution and repair. Biol Chem. (2016) 397:971–9. 10.1515/hsz-2016-0180 27232634 PMC5361892

[35] Chatterjee BE Yona S Rosignoli G Young RE Nourshargh S Flower RJ. Annexin 1-deficient neutrophils exhibit enhanced transmigration *in vivo* and increased responsiveness *in vitro*. J Leukoc Biol. (2005) 78:639–46. 10.1189/jlb.0405206 16000391

[36] Getting SJ Flower RJ Perretti M. Inhibition of neutrophil and monocyte recruitment by endogenous and exogenous lipocortin 1. Br J Pharmacol. (1997) 120:1075–82. 10.1038/sj.bjp.0701029 9134220 PMC1564582

[37] Gavins FNE Hickey MJ. Annexin A1 and the regulation of innate and adaptive immunity. Front Immunol. (2012) 3:354. 10.3389/fimmu.2012.00354 23230437 PMC3515881

[38] Dalli J Montero-Melendez T McArthur S Perretti M. Annexin A1 N-terminal derived Peptide ac2-26 exerts chemokinetic effects on human neutrophils. Front Pharmacol. (2012) 3:28. 10.3389/fphar.2012.00028 22403546 PMC3288723

[39] Damazo AS Yona S Flower RJ Perretti M Oliani SM. Spatial and temporal profiles for anti-inflammatory gene expression in leukocytes during a resolving model of peritonitis. J Immunol. (2006) 176:4410–8. 10.4049/JIMMUNOL.176.7.4410 16547279 PMC1868080

[40] Zanardo RCO Perretti M Wallace JL. Annexin-1 is an endogenous gastroprotective factor against indomethacin-induced damage. Am J Physiol Liver Physiol. (2005) 288:G481–6. 10.1152/ajpgi.00299.2004 15472012

[41] Solito E de Coupade C Canaider S Goulding NJ Perretti M. Transfection of annexin 1 in monocytic cells produces a high degree of spontaneous and stimulated apoptosis associated with caspase-3 activation. Br J Pharmacol. (2001) 133:217–28. 10.1038/sj.bjp.0704054 11350857 PMC1572776

[42] McArthur S Gobbetti T Kusters DHM Reutelingsperger CP Flower RJ Perretti M. Definition of a novel pathway centered on lysophosphatidic acid to recruit monocytes during the resolution phase of tissue inflammation. J Immunol. (2015) 195:1139–51. 10.4049/jimmunol.1500733 26101324 PMC4505961

[43] Maderna P Yona S Perretti M Godson C. Modulation of phagocytosis of apoptotic neutrophils by supernatant from dexamethasone-treated macrophages and annexin-derived peptide Ac(2-26). J Immunol. (2005) 174:3727–33. 10.4049/jimmunol.174.6.3727 15749912

[44] Dalli J Jones CP Cavalcanti DM Farsky SH Perretti M Rankin SM. Annexin A1 regulates neutrophil clearance by macrophages in the mouse bone marrow. FASEB J. (2012) 26:387–96. 10.1096/fj.11-182089 21957127 PMC3250241

[45] McArthur S Loiola RA Maggioli E Errede M Virgintino D Solito E. The restorative role of annexin A1 at the blood–brain barrier. Fluids Barriers CNS. (2016) 13:17. 10.1186/s12987-016-0043-0 27655189 PMC5031267

[46] Probst-Cousin S Kowolik D Kuchelmeister K Kayser C Neundörfer B Heuss D. Expression of annexin-1 in multiple sclerosis plaques. Neuropathol Appl Neurobiol. (2002) 28:292–300. 10.1046/j.1365-2990.2002.00396.x 12175341

[47] Ries M Loiola R Shah UN Gentleman SM Solito E Sastre M. The anti-inflammatory Annexin A1 induces the clearance and degradation of the amyloid-β peptide. J Neuroinflammation. (2016) 13:234. 10.1186/s12974-016-0692-6 27590054 PMC5010757

[48] Solito E Sastre M. Microglia function in Alzheimer's Disease. Front Pharmacol. (2012) 3:14. 10.3389/fphar.2012.00014 22363284 PMC3277080

[49] Kusters DHM Chatrou ML Willems BAG De Saint-Hubert M Bauwens M van der Vorst E Pharmacological treatment with annexin A1 reduces atherosclerotic plaque burden in LDLR^−/−^ mice on western type diet. PLoS ONE. (2015) 10:e0130484 10.1371/journal.pone.0130484 26090792 PMC4475013

[50] Petri MH Laguna-Fernández A Gonzalez-Diez M Paulsson-Berne G Hansson GK Bäck M. The role of the FPR2/ALX receptor in atherosclerosis development and plaque stability. Cardiovasc Res. (2015) 105:65–74. 10.1093/cvr/cvu224 25341894 PMC4277257

[51] Drechsler M de Jong R Rossaint J Viola JR Leoni G Wang JM. Annexin A1 counteracts chemokine-induced arterial myeloid cell recruitment. Circ Res. (2015) 116:827–35. 10.1161/CIRCRESAHA.116.305825 25520364 PMC7751381

[52] Fredman G Kamaly N Spolitu S Milton J Ghorpade D Chiasson R. Targeted nanoparticles containing the proresolving peptide Ac2-26 protect against advanced atherosclerosis in hypercholesterolemic mice. Sci Transl Med. (2015) 7:275ra20. 10.1126/scitranslmed.aaa1065 25695999 PMC4397585

[53] de Jong RJ Paulin N Lemnitzer P Viola JR Winter C Ferraro B. Protective aptitude of annexin A1 in arterial neointima formation in atherosclerosis-prone mice—brief report. Arterioscler Thromb Vasc Biol. (2017) 37:312–5. 10.1161/ATVBAHA.116.308744 28062503

[54] Gobbetti T Coldewey SM Chen J McArthur S le Faouder P Cenac N. Nonredundant protective properties of FPR2/ALX in polymicrobial murine sepsis. Proc Natl Acad Sci USA. (2014) 111:18685–90. 10.1073/pnas.1410938111 25512512 PMC4284560

[55] Bergström I Lundberg AK Jönsson S Särndahl E Ernerudh J Jonasson L. Annexin A1 in blood mononuclear cells from patients with coronary artery disease: its association with inflammatory status and glucocorticoid sensitivity. PLoS ONE. (2017) 12:e0174177. 10.1371/journal.pone.0174177 28329022 PMC5362084

[56] Ansari J Kaur G Gavins FNE. Therapeutic potential of annexin A1 in ischemia reperfusion injury. Int J Mol Sci. 19:E1211. 10.3390/ijms19041211 29659553 PMC5979321

[57] D'amico M Di Filippo C La M Solito E Mclean PG Flower RJ. Lipocortin 1 reduces myocardial ischemia-reperfusion injury by affecting local leukocyte recruitment. FASEB J. (2000) 14:1867–9. 10.1096/fj.99-0602fje 11023969

[58] La M D'Amico M Bandiera S Di Filippo C Oliani SM Gavins FN. Annexin 1 peptides protect against experimental myocardial ischemia-reperfusion: analysis of their mechanism of action. FASEB J. (2001) 15:2247–56. 10.1096/fj.01-0196com 11641252

[59] Ritchie RH Gordon JM Woodman OL Cao AH Dusting GJ. Annexin-1 peptide Anx-1(2-26) protects adult rat cardiac myocytes from cellular injury induced by simulated ischaemia. Br J Pharmacol. (2005) 145:495–502. 10.1038/sj.bjp.0706211 15821756 PMC1576163

[60] Qin CX May LT Li R Cao N Rosli S Deo M. Small-molecule-biased formyl peptide receptor agonist compound 17b protects against myocardial ischaemia-reperfusion injury in mice. Nat Commun. (2017) 8:14232. 10.1038/ncomms14232 28169296 PMC5309721

[61] Relton JK Strijbos PJ O'Shaughnessy CT Carey F Forder RA Tilders FJ. Lipocortin-1 is an endogenous inhibitor of ischemic damage in the rat brain. J Exp Med. (1991) 174:305–10. 1830327 10.1084/jem.174.2.305 PMC2118906

[62] Smith HK Gil CD Oliani SM Gavins FNE. Targeting formyl peptide receptor 2 reduces leukocyte-endothelial interactions in a murine model of stroke. FASEB J. (2015) 29:2161–71. 10.1096/fj.14-263160 25690650

[63] Vital SA Becker F Holloway PM Russell J Perretti M Granger DN Formyl-peptide receptor 2/3/lipoxin A _4_ receptor regulates neutrophil-platelet aggregation and attenuates cerebral inflammation. Circulation. (2016) 133:2169–79. 10.1161/CIRCULATIONAHA.115.020633 27154726 PMC4889496

[64] Zhao B Wang J Liu L Li X Liu S Xia Q. Annexin A1 translocates to nucleus and promotes the expression of pro-inflammatory cytokines in a PKC-dependent manner after OGD/R. Sci Rep. (2016) 6:27028. 10.1038/srep27028 27426034 PMC4947919

[65] Melki V Hullin F Mazarguil H Fauvel J Ragabthomas JMF Chap H. Annexin I as a potential inhibitor of insulin receptor protein tyrosine kinase. Biochem Biophys Res Commun. (1994) 203:813–9. 10.1006/bbrc.1994.2255 8093061

[66] Ohnishi M Tokuda M Masaki T Fujimura T Tai Y Itano T. Involvement of annexin-I in glucose-induced insulin secretion in rat pancreatic islets. Endocrinology. (1995) 136:2421–6. 10.1210/endo.136.6.7750463 7750463

[67] Rackham CL Vargas AE Hawkes RG Amisten S Persaud SJ Austin ALF. Annexin A1 is a key modulator of mesenchymal stromal cell mediated improvements in islet function. Diabetes. (2015) 65:db150990. 10.2337/db15-0990 26470781

[68] Purvis GSD Chiazza F Collino M Solito E Thiemermann C Endogenous annexin-A1 is a protective determinant in HFD-induced insulin resistance and diabetic nephropathy. FASEB J. (2017) 31(1_Suppl.):853.3 10.1096/fasebj.31.1_supplement.853.328246297

[69] Purvis GSD Collino M Azevedo Loiola R Baragetti A Chiazza F Brovelli M. Identification of AnnexinA1 as an endogenous regulator of RhoA, and its role in the pathophysiology and experimental therapy of type 2 diabetes. Front Immunol. (2019) 10:571. 10.3389/FIMMU.2019.00571 30972066 PMC6446914

[70] Kosicka A Cunliffe AD Mackenzie R Zariwala MG Perretti M Flower RJ. Attenuation of plasma annexin A1 in human obesity. FASEB J. (2013) 27:368–78. 10.1096/fj.12-213728 23038751

[71] Yoon JH Kim D Jang J-H Ghim J Park S Song P. Proteomic analysis of the palmitate-induced myotube secretome reveals involvement of the annexin A1-formyl peptide receptor 2 (FPR2) pathway in insulin resistance. Mol Cell Proteomics. (2015) 14:882–92. 10.1074/mcp.M114.039651 25616869 PMC4390267

[72] Begum N Sandu OA Ito M Lohmann SM Smolenski A. Active rho kinase (ROK-α) associates with insulin receptor substrate-1 and inhibits insulin signaling in vascular smooth muscle cells. J Biol Chem. (2002) 277:6214–22. 10.1074/jbc.M110508200 11739394

[73] Pietrani NT Ferreira CN Rodrigues KF Perucci LO Carneiro FS Bosco AA. Proresolving protein annexin A1: the role in type 2 diabetes mellitus and obesity. Biomed Pharmacother. (2018) 103:482–9. 10.1016/j.biopha.2018.04.024 29677533

[74] Ka S-M Tsai P-Y Chao T-K Yang S-M Hung Y-J Chen J-S. Urine annexin A1 as an index for glomerular injury in patients. Dis Markers. (2014) 2014:854163. 10.1155/2014/854163 24591769 PMC3925619

[75] Vithian K Hurel S. Microvascular complications: pathophysiology and management. Clin Med. (2010) 10:505–9. 10.7861/CLINMEDICINE.10-5-505 21117389 PMC4952418

[76] Maeda M Hayashi T Mizuno N Hattori Y Kuzuya M. Intermittent high glucose implements stress-induced senescence in human vascular endothelial cells: role of superoxide production by NADPH oxidase. PLoS ONE. (2015) 10:e0123169. 10.1371/journal.pone.0123169 25879533 PMC4400006

[77] Leung WK Gao L Siu PM Lai CW. Diabetic nephropathy and endothelial dysfunction: current and future therapies, and emerging of vascular imaging for preclinical renal-kinetic study. Life Sci. (2016) 166:121–30. 10.1016/J.LFS.2016.10.015 27765534

[78] Jelinic M Deo M Finlayson S Kahlberg N Kiriazis H Du X Annexin-A1^−/−^ Mice with type 2 diabetes exhibit cardiovascular dysfunction and exaggerated inflammation despite less pronounced hyperglycaemia. Hear Lung Circ. (2018) 27:S76 10.1016/j.hlc.2018.06.076

[79] Qiao Y-N He W-Q Chen C-P Zhang C-H Zhao W Wang P. Myosin phosphatase target subunit 1 (MYPT1) regulates the contraction and relaxation of vascular smooth muscle and maintains blood pressure. J Biol Chem. (2014) 289:22512–23. 10.1074/jbc.M113.525444 24951589 PMC4139257

[80] Neymeyer H Labes R Reverte V Saez F Stroh T Dathe C. Activation of annexin A1 signalling in renal fibroblasts exerts antifibrotic effects. Acta Physiol. (2015) 215:144–58. 10.1111/apha.12586 26332853

[81] Labes R Dittert P Bachmann S Paliege A Sources for anti-inflammatory annexin A1 signals during acute anti-Thy-1.1 nephritis. FASEB J. (2016) 30(1_Suppl.):1217.15.

[82] Qin C Yang YH May L Gao X Stewart AG Tu Y. Cardioprotective potential of annexin-A1 mimetics in myocardial infarction. Pharmacol Ther. (2014) 148:47–65. 10.1016/j.pharmthera.2014.11.012 25460034

[83] Locatelli I Sutti S Jindal A Vacchiano M Bozzola C Reutelingsperger C. Endogenous annexin A1 is a novel protective determinant in nonalcoholic steatohepatitis in mice. Hepatology. (2014) 60:531–44. 10.1002/hep.27141 24668763 PMC4258084

[84] Yamada N Martin LB Zechendorf E Purvis GSD Chiazza F Varrone B. Novel synthetic, host-defense peptide protects against organ injury/dysfunction in a rat model of severe hemorrhagic shock. Ann Surg. (2017) 268:348–56. 10.1097/SLA.0000000000002186 28288070

